# Direct Transcriptional Control of a p38 MAPK Pathway by the Circadian Clock in *Neurospora crassa*

**DOI:** 10.1371/journal.pone.0027149

**Published:** 2011-11-07

**Authors:** Teresa M. Lamb, Charles S. Goldsmith, Lindsay Bennett, Katelyn E. Finch, Deborah Bell-Pedersen

**Affiliations:** Program for the Biology of Filamentous Fungi, Interdisciplinary Program in Genetics, Department of Biology, Center for Biological Clocks Research, Texas A&M University, College Station, Texas, United States of America; Cinvestav, Mexico

## Abstract

MAPK signal transduction pathways are important regulators of stress responses, cellular growth, and differentiation. In Neurospora, the circadian clock controls rhythms in phosphorylation of the p38-like MAPK (OS-2); however, the mechanism for this regulation is not known. We show that the WCC, a transcription factor and clock component, binds to the *os-4* MAPKKK promoter in response to light and rhythmically in constant darkness, peaking in the subjective morning. Deletion of the WCC binding sites in the *os-4* promoter disrupts both *os-4* mRNA and OS-2 phosphorylation rhythms. The clock also indirectly regulates rhythmic expression of the histidyl-phosphotransferase gene, *hpt-1*, which peaks in the evening. Anti-phase expression of positive (OS-4) and negative (HPT-1) MAPK pathway regulators likely coordinate to enhance rhythmic MAPK activation to prepare cells to respond to osmotic stress during the day in the natural environment. Consistent with this idea, we show that wild type cells have a clock-dependent morning kinetic advantage in glycerol accumulation after salt stress as compared to evening treatment. Thus, circadian transcriptional control of MAPK pathway components leads to striking time-of-day-specific effects on the signaling status and physiological response of the pathway.

## Introduction

Eukaryotic cells adapt their physiology to respond to changing extracellular conditions for enhanced fitness and survival. Two complex mechanisms exist in cells to deal with environmental conditions: adaptation and anticipation. When the environment changes in unpredictable ways, conserved stress-induced mitogen-activated protein kinase (MAPK) signaling pathways are activated to promote adaptation [1]. The acute response mobilized by the MAPK pathways can occur at any time of day to improve survival [2]. When the environment changes on a predictable basis, such as the day/night cycle, an internal timing mechanism called the circadian clock provides the machinery to anticipate the change and program daily rhythms in gene expression to prepare for daily stresses. In this way, the clock provides an adaptive advantage to organisms [3], [4]. Because these two mechanisms can be directed to cope with similar stressors, for example light, heat, or osmotic stress, linkage of the two would maximize cellular efficiency, and evidence for such a linkage exists in *Neurospora crassa* [2], [5]. The Neurospora circadian clock directs the rhythmic phosphorylation, and activation, of the osmotic sensing (OS)-pathway p-38-like MAPK (OS-2) [2]. The mechanistic connection between the clock and the stress responsive MAPK pathway is unknown, and critical to understand how organisms prepare for daily predictable stresses. Furthermore, defining this link is an important step in understanding how defects in circadian clocks and defects in MAPK pathways cause similar diseases in humans, including immune system dysfunction, heart disease, neurodegenerative disorders, and cancer [6], [7], [8], [9].

The OS-pathway of Neurospora is a conserved pathway closely related to the *S. pombe* Sty1 pathway and similar to the highly-characterized HOG pathway of *S. cerevisiae*, that senses and responds to multiple environmental stresses, but is most noted for its role in the hyper-osmotic shock response [10], [11], [12], [13], [14]. Osmotic activation of this pathway ultimately results in the production of small molecules that adjust internal osmotic pressure [13], [15], [16], [17], [18]. Input to the Neurospora OS pathway involves a phosphorelay whereby a sensor histidine kinase (OS-1) detects an environmental signal, which is propagated through a histidine phosphotransferase (HPT-1) to a response regulator (RRG-1) [10]. RRG-1 modulates the activity, by an unknown mechanism, of a MAPK cascade that includes OS-4 (MAPKKK), OS-5 (MAPKK), and the p-38-like OS-2 (MAPK) [12], [19]. Activated MAPK regulates the activities of effector molecules, including transcription factors, other kinases, translation factors, and chromatin remodeling proteins [20], [21], [22]. In Neurospora, these effectors are thought to control downstream targets that encode components needed to survive conditions of high osmolarity, as well as for conidial integrity, sexual development, and fungicide sensitivity [10], [23], [24]. Similarly in mammals, the p38 family of stress activated MAPKs (SAPKs) are activated by a variety of extracellular stimuli including UV light, heat shock, osmotic stress, inflammatory cytokines, and the internal circadian clock [25]. Activated mammalian p38 MAPK controls the expression of more than 100 different genes, such as those involved cell proliferation, apoptosis, and tumor suppression [26], [27]. Thus, understanding how the clock regulates the OS MAPK pathway in Neurospora will be informative for understanding this connection in higher organisms.

The circadian clock system that allows organisms to anticipate predictable changes in the environment is composed of endogenous molecular oscillators that function to generate a free-running period that is close to 24-h when the organism is kept in constant environmental conditions, and an exactly 24-h period in natural environmental cycles [28]. The oscillators comprise the products of “clock genes” that are organized in transcriptional-translational feedback loops [29], [30]. These clock genes encode transcription activators and negative elements that feedback to inhibit their own expression by disrupting the activity of the activators. Components of the oscillators receive environmental information through input pathways, allowing the oscillators to remain synchronized to the 24-h solar day [31], [32].

Time-of-day information from the oscillator(s) is relayed through output pathways to control expression of the clock-controlled genes (ccgs) and overt rhythmicity. One mechanism by which the output pathways are controlled is through the rhythmic activity of transcription factors that are themselves components of the oscillator. For example, in mammals, the positive oscillator components mCLK and BMAL1 bind to E-box elements in the promoters of some clock outputs including *Dbp* and *Avp*, thereby driving rhythmic transcription [33], [34], [35], [36], [37].

In the well-characterized Neurospora FRQ/WCC oscillator (FWO), the positive oscillator components WHITE COLLAR-1 (WC-1) and WC-2 dimerize to form the white collar complex (WCC) [38]. The WCC functions as a blue light photoreceptor and as a transcription factor in the core oscillator to turn on expression of *frq* encoding the negative element FREQUENCY (FRQ) [39], [40], [41], [42]. In addition to the role of the WCC in photoresponses and in the oscillator, the WCC signals time of day information directly to downstream ccgs [2], [22], [43], [44]. In a recent study, Smith *et al* (2010) demonstrated that the WCC binds to hundreds of genomic regions, including the promoters of previously identified clock- and light-regulated genes, as well as a suite of second tier transcription factors and signaling molecules.

We previously demonstrated that the Neurospora OS pathway functions as an output pathway from the FWO [2], [6]. Under constant environmental conditions, time-of-day information is somehow transferred from the FWO resulting in rhythms in OS-2 phosphorylation. OS-2 phosphorylation levels peak in the early subjective morning and are at the lowest in the night. This would allow the cells to be prepared for daily daytime stress, including light, heat, and desiccation. However, the FWO is not required for Neurospora cells to mount an acute response to osmotic shock; FRQ or WC-1 deletion strains show rapid phosphorylation of OS-2 following a salt shock [2]. This suggests that the OS pathway receives information from at least two sources: the endogenous clock and the external environment. While environmental input to MAPK pathways has been well studied [13], [45], how the endogenous clock signal is perceived by the MAPK pathway is not known.

In this study, we investigated the mechanisms by which the clock regulates rhythmic activity of the OS pathway. We found that the clock- and light-associated WCC directly regulates the MAPK pathway through rhythmic binding to the promoter of the MAPKKK gene *os-4*, resulting in rhythmic *os-4* transcription and OS-4 protein accumulation. We demonstrate that deletion of the *os-4* WCC binding sites abolishes rhythmic expression of *os-4*, disrupts rhythmic accumulation of phospho-OS-2, and abolishes circadian clock-dependent effects on the kinetics of glycerol accumulation. Additionally, a component of the phosphorelay, *hpt-1*, is shown to be clock-regulated, with mRNA levels peaking at a phase opposite of *os-4* mRNA. This antiphase regulation of the phosphorelay and MAPK module by the clock likely contributes to the robustness of the rhythm in OS-2 activity. Whereas the major focus on the regulation of MAPK pathways has been at the level of posttranslational control of phosphorylation, our results suggest an important role for transcription initiation in the regulation of MAPK pathway components and signaling. Together, these findings may have important implications in treatments for diseases associated with defective MAPK pathways.

## Results

### The promoter of the *os-4* gene, encoding a MAPKKK, is a direct target of the WCC

**Figure 1:**
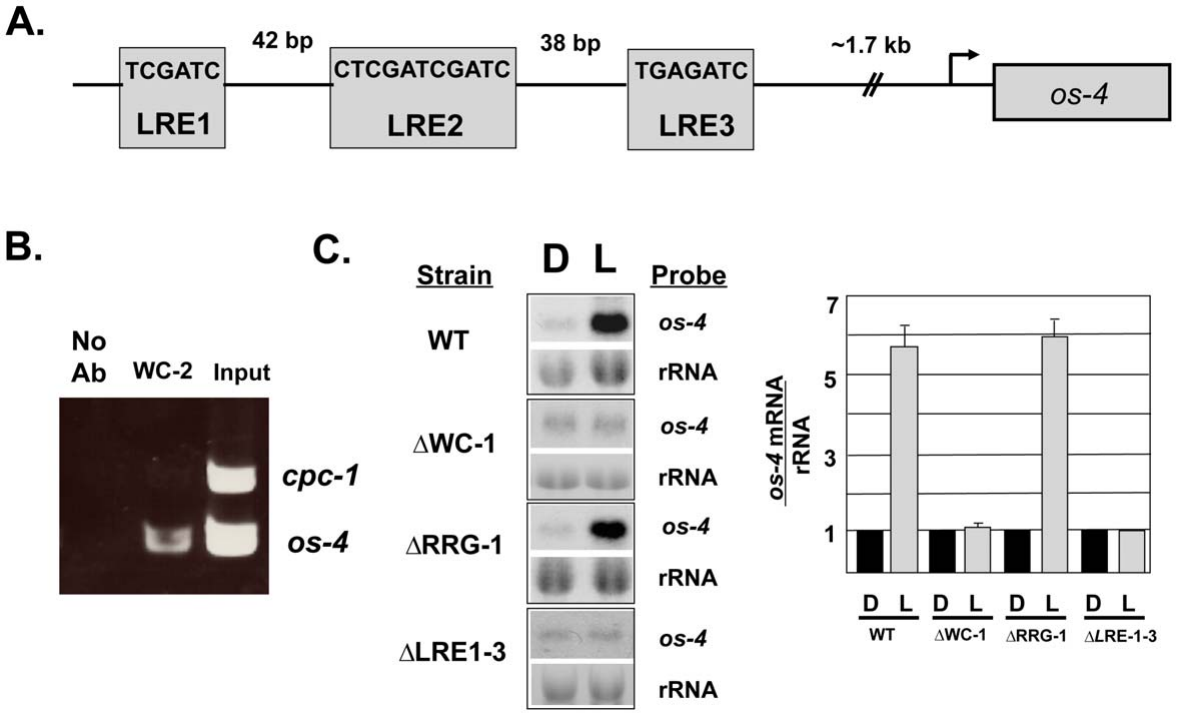
WC-2 binds to the *os-4* promoter and binding is necessary for light induction of *os-4* mRNA. (**A**) Candidate WCC binding sites within the *os-4* promoter region. The sequences of three candidate light-responsive elements (LREs) that lie within the 500 bp region of the WCC binding site, about 1.7 kb upstream of the start site of transcription are shown. The size of the drawing is not to scale. (**B**) ChIP-PCR assay using WC-2 antibody showing binding to the *os-4* promoter, but not to the *cpc-1* gene, following a 10-min light pulse of cultures. Input DNA and no antibody (no Ab) are shown as controls. (**C**) Representative northern blot analysis of *os-4* mRNA from cultures grown in the dark (D) or given a 30-min light pulse (L) in the indicated strains. rRNA is shown as a loading control. Densitometry analysis of northern blot experiments (n = 4 ± SEM) is shown on the right.

To characterize the output pathways from the FWO, we previously carried out a comprehensive ChIP-seq study to identify the direct targets of the WCC using antibody directed against WC-1 and WC-2 [44]. The cultures were given an 8-min light pulse prior to ChIP to activate the WCC; thus, we identified the top tier genes involved in light signaling pathways and circadian clock output pathways. Using this method, hundreds of direct targets of the WCC were identified, most of which were present in the promoters of genes, including a 500 bp region (nt 4448839–4449339 of chromosome 1) that resides about 1.7 kb upstream of the predicted start of transcription for the *os-4* gene. Within this 500 bp region of the *os-4* promoter, 3 candidate binding sites (called light-responsive elements [LRE] 1–3) for the WCC were identified that closely match a consensus binding site (GATCGA) derived from the ChIP-seq target data for the WCC [44] (Figure 1A).

To validate the WCC ChIP-seq results for the *os-4* promoter, an independent replicate WC-2 ChIP, followed by region-specific PCR from cultures given a light pulse was carried out (Figure 1B). Enrichment of WC-2 binding was observed in the same *os-4* promoter region revealed by ChIP-seq, but as expected, not in the control *cpc-1* gene which lacks a WCC binding site.

Of the direct targets of the WCC, most, but not all, genes are light-induced [44]. To determine if *os-4* expression is photoinduced, the level of *os-4* mRNA was measured in cells before and after a light pulse. We found that a 30-min light pulse induced *os-*4 transcript levels by about 6-fold, and that WC-1, but not RRG-1 (a component of the phosphorelay), is necessary for this induction (Figure 1C).

Together, these data demonstrate that the WCC binds to the *os-4* promoter and activates *os-4* transcription in response to light. Consistent with this result, regulation of *os-4* by light occurs independently of a functional phosphorelay required for induction of the MAPK pathway by an acute osmotic shock [10].

### The *os-4* gene is regulated by the circadian clock

**Figure 2:**
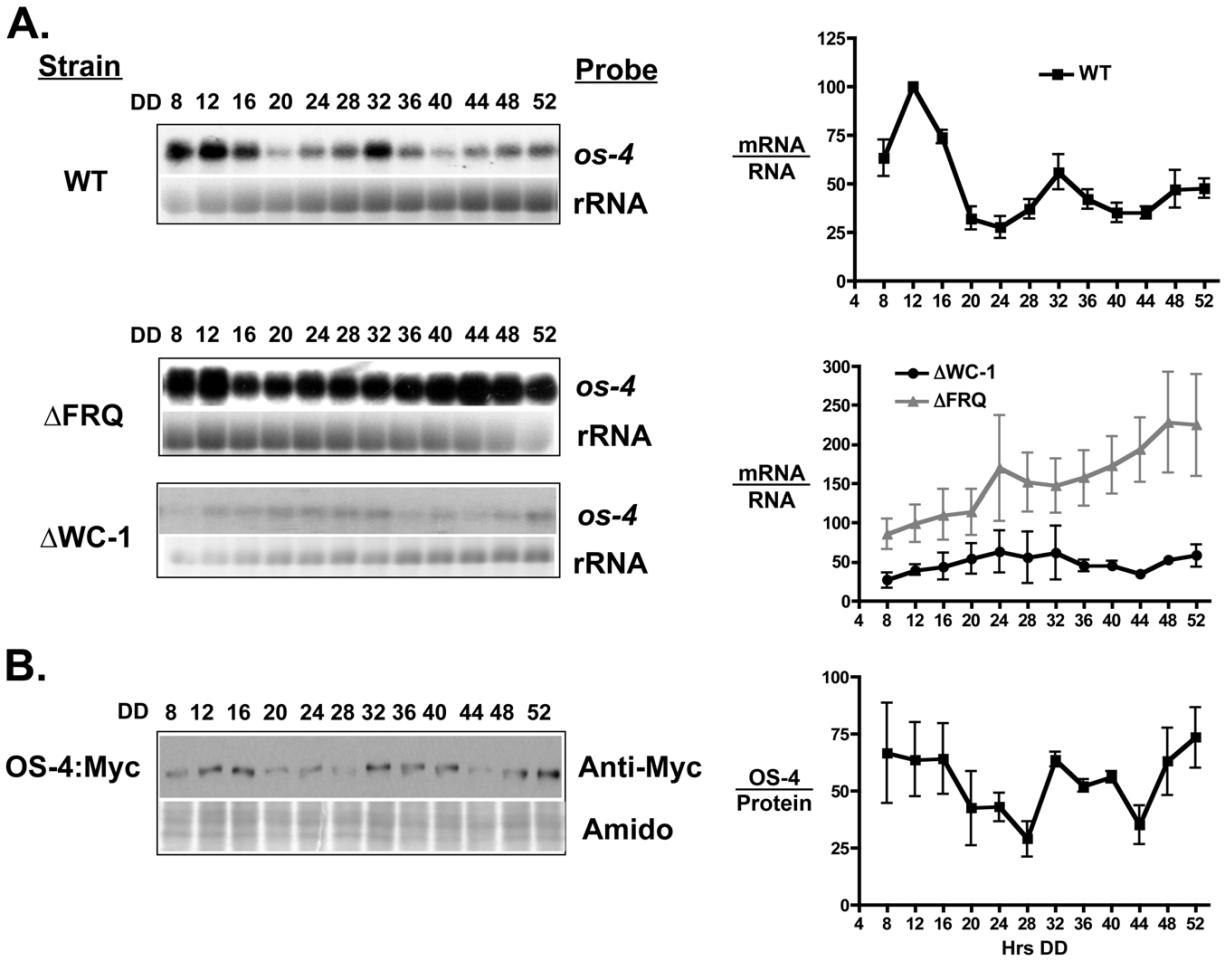
The *os-4* gene is expressed with a circadian rhythm. (**A**) Representative northern blots showing *os-4* mRNA levels in the indicated strains from cultures grown in the dark (DD) and harvested every 4-h over the course of two days (left). rRNA is shown as a loading control. Densitometric analysis of northern blot experiments are plotted and shown on the right (n = 3 ± SEM). The average peak in mRNA accumulation in WT cells for each replicate experiment was set to 100. The data for ΔWC-1 and ΔFRQ are normalized to *os-4* levels in the WT strain making the levels directly comparable to each other. (**B**) Representative western blot using Myc antibody to detect the OS-4:Myc fusion protein from Neurospora cells grown as in A (left). Amido black stained protein is shown below as a loading control. Densitometric analysis of western blot experiments are plotted and shown on the right (n = 3 ± SEM). The average peak in protein accumulation in WT cells for each replicate experiment was set to 100.

The WCC functions as a positive element in the FWO by binding to the *frq* promoter and activating transcription in the morning [46], [47] leading to rhythms in FRQ protein. Thus, the discovery that the WCC binds to the promoter of the *os-4* gene suggested the possibility that *os-4* is expressed with a circadian rhythm. To test this idea, WT clock cells were grown in constant dark (DD) for 2 days, harvested every 4 h, and RNA isolated from the cells was probed with an *os-4-*specific probe. A robust circadian rhythm in *os-4* mRNA accumulation was observed (p≤0.007; period 21.5±0.6 h), with peaks occurring in the subjective morning at 12 and 32 h in DD (Figure 2). As expected for a gene regulated by the FWO, this rhythm was abolished in strains deleted for either FRQ or WC-1. The levels of *os-4* transcripts were generally low in the ΔWC-1 strain, and high in the ΔFRQ strain, as compared to the WT strain. These data are consistent with direct activation of *os-4* transcription by the WCC, and regulation of *os-4* by the FWO, whereby the loss of FRQ in cells results in constitutively active WCC and high levels of *os-4* mRNA. The *os-4* mRNA rhythms persisted in an ΔRRG-1 strain (Figure S1), indicating that similar to the light response, RRG-1 is not necessary for *os-4* mRNA rhythms. Taken together, these data suggest that clock and light input into the MAPK module occurs at the level of *os-4* transcription.

To determine if accumulation of OS-4 protein is similarly rhythmic, the endogenous *os-4* gene was replaced with an OS-4:7xMyc-tagged construct, and accumulation of the tagged protein was examined over the course of the day. The tagged protein retained normal activity, as the tagged strain was resistant to high salt conditions that are lethal to an *os-4* deletion strain (data not shown). A statistically significant, low amplitude rhythm was observed in OS-4::7xMyc protein (p≤0.03; period 20.4±1.4 h), with peaks occurring in the subjective morning, similar to *os-4* mRNA peaks (Figure 2B).

### WC-2 binds rhythmically to the *os-4* promoter

**Figure 3:**
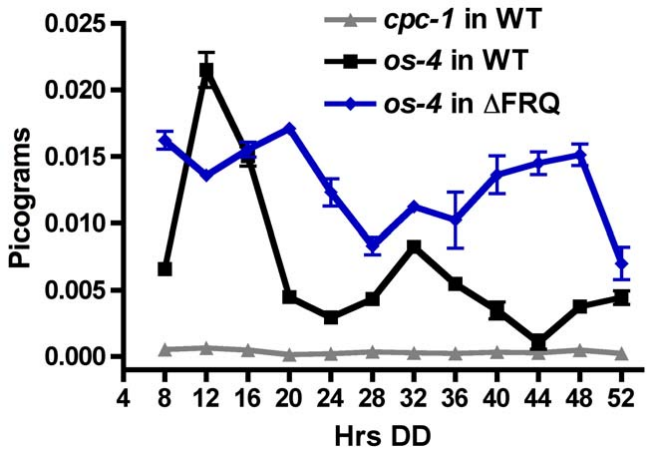
The WCC binds rhythmically to the *os-4* promoter. Plot of ChIP-quantitative PCR results for the *os-4* promoter in WT (black line and squares) and Δ*frq* (blue lines and diamonds) strains, and the control *cpc-1* gene in a WT (grey line and triangles) at the indicated times in the dark (Hrs DD) (n = 4 sample replicates ± SEM). Two biological replicates yielded similar results.

We have shown that the WCC binds to the promoter of *os-4*, and that *os-4* mRNA accumulates rhythmically, peaking in the morning at the time of day when the WCC is most active [48]. To determine if rhythmic expression of *os-4* is due to WCC binding to the promoter at specific times of the day, WC-2 ChIP/qPCR was carried out on cultures grown in DD and harvested every 4 h over two days. For WT clock cells, an enhanced enrichment of *os-4* promoter DNA associating with the WCC in the morning samples (DD12 and DD32), and reduced enrichment in evening samples (DD24 and DD44), was observed (Figure 3). This is consistent with the morning-specific expression of *os-4* mRNA (Figure 2). In the ΔFRQ strain, *os-4* promoter DNA associated with the WCC generally at all times of day (Figure 3), coincident with constitutive high levels of *os-4* mRNA in ΔFRQ cells (Figure 2). As a control, PCR for *cpc-1*, a gene not regulated by the WCC, was conducted. As expected, low levels of the *cpc-1* PCR product were detected at all times of day (Figure 3). These observations revealed that the WCC rhythmically binds to the *os-4* promoter, and that rhythmic WCC binding to the *os-4* promoter requires FRQ.

### Rhythmic transcription of *os-4* affects rhythmic activity of the MAPK pathway

**Figure 4:**
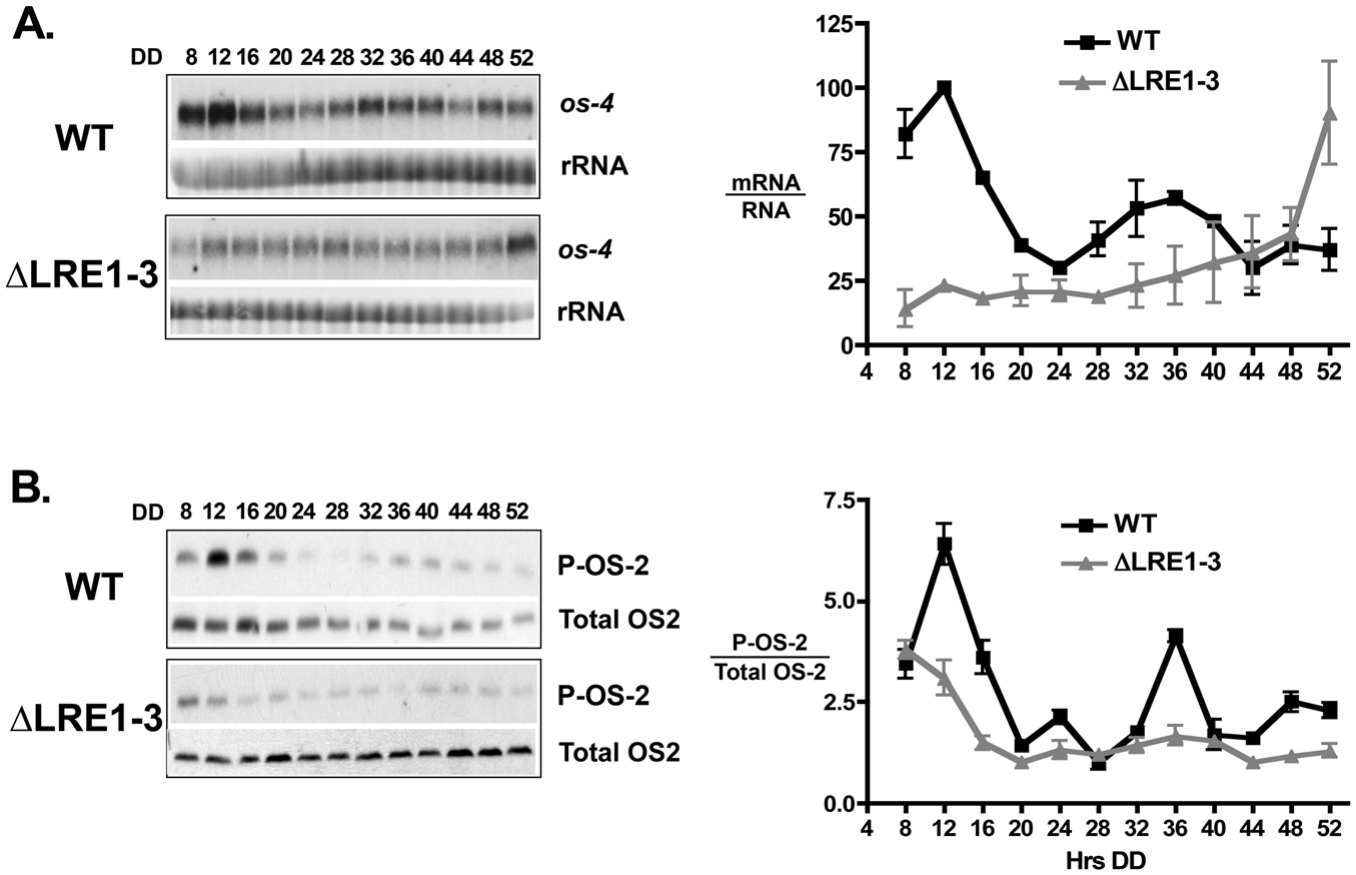
Deletion of the WCC binding site on the *os-4* promoter abolishes rhythms in *os-4* mRNA and phospho-OS-2 accumulation. (**A**) Northern blot assay of *os-4* mRNA from WT (top) and ΔLRE1-3-*os-4* cells (ΔLRE1-3) harvested at the indicated times in the dark (DD). rRNA serves as a loading control. Densitometric analyses of the northern blot experiments are plotted on the right as the level of *os-4* mRNA over rRNA (n = 3 ± SEM). The peak levels *os-4* mRNA in WT were set to 100 for each experiment, and the data for ΔLRE1-3 are normalized to the peak *os-4* mRNA levels in the WT strain making the levels directly comparable to each other. (**B**) Western blot of protein isolated from WT and ΔLRE1-3-*os-4* (ΔLRE1-3) cells and probed with phospho-specific p38 antibody (P-OS-2) or an antibody that recognized phosphorylated and un-phosphorylated OS-2 (total OS-2). Densitometric analysis of the western blot experiments are plotted as the level of P-OS-2 over total OS-2 protein, and shown on the right (n = 4 ± SEM).

Stress signaling through MAPK pathways results in phosphorylation of existing MAPK protein components. However, very little is known about the effects of transcriptional regulation on signaling. We have shown that the circadian clock regulates rhythmic transcriptional activation of *os-4* through rhythmic binding of the WCC to the promoter. This activity leads to rhythmic accumulation of OS-4 protein. We then asked if rhythmic transcription of *os-4* is required for rhythms in phosphorylation of the downstream p-38-like MAPK (OS-2). As OS-2 phosphorylation is undetectable in an *os-4* deletion strain [49], an alternate way of disrupting the rhythmicity of *os-4* expression was required. Based on the failed light induction of *os-4* in the *Δ*LRE1-3-*os-4* promoter mutant strain (Figure 1C), we predicted that this strain might also have defects in *os-4* mRNA rhythms. Figure 4A shows that the ΔLRE1-3-*os4* strain still maintains expression of *os-4* mRNA, but that it is not rhythmic. Thus, we tested whether the rhythms in *os-4* transcripts are required for the phosphorylation rhythms in OS-2 using the ΔLRE1-3-*os-4* strain. Deletion of the LREs in the *os-4* promoter reduced the absolute levels of phospho-OS-2 (Figure 4B). Despite this reduction in phospho-OS-2 levels, the amount produced was sufficient to render the strains resistant to salt treatment (data not shown). In addition, OS-2 phosphorylation and *os-4* mRNA were both induced by treatment of ΔLRE1-3-*os-4* cells with 4% NaCl (Figure S2 and S3, respectively), supporting the idea that the clock and the environment use independent pathways for activation of OS-2, and that the *ΔLRE1-3-os4* strain has no defect in stress responses. In WT strains, phospho-OS-2 accumulated with a circadian rhythm (p≤0.008, period 22.8±1.4 h), while deletion of the LREs in the *os-4* promoter disrupted the normal rhythms in phospho-OS-2 accumulation (Figure 4B). Taken together, these data demonstrate that direct activation of *os-4* transcription by the WCC in the morning is necessary for robust rhythms in OS-2 phosphorylation in constant environmental conditions.

### Evening-specific regulation of *hpt-1* mRNA levels by the circadian clock

**Figure 5:**
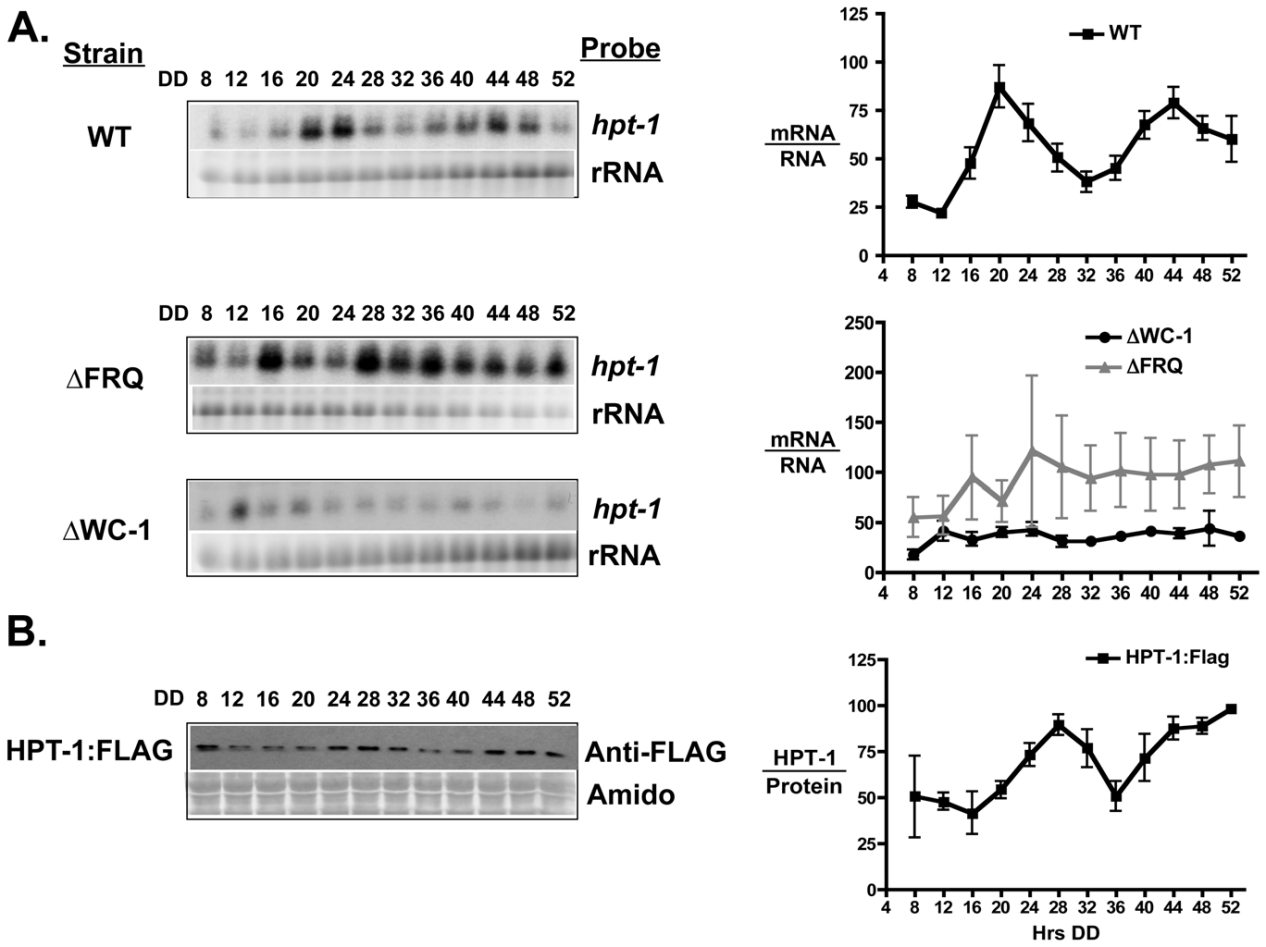
The *hpt-1* gene is expressed with a circadian rhythm. (**A**) Representative northern blots showing *hpt-1* mRNA levels in the indicated strains from cultures grown in the dark (DD) and harvested every 4-h over the course of two days (left). rRNA is shown as a loading control. Densitometric analysis of northern blot experiments are plotted and shown on the right (n = 3 ± SEM). The average peak in *hpt-1* mRNA accumulation in WT cells for each replicate experiment was set to 100. The data for ΔWC-1 and ΔFRQ are normalized to *hpt-1* levels in the WT strain making the levels directly comparable to each other. (**B**) Representative western blot using FLAG antibody to detect the HPT-1:FLAG fusion protein from Neurospora cells grown as in A (left). Amido stained protein is shown below as a loading control. Densitometric analysis of western blot experiments are plotted and shown on the right (n = 3 ± SEM). The average peak in protein accumulation in WT cells for each replicate experiment was set to 100.

To determine if the circadian clock regulates the expression of other OS pathway components, each of the pathway components was assayed for rhythmicity. No statistically significant rhythm was observed in total protein accumulation for OS-2 (Figure 4) [2], or in mRNA accumulation for the histidine kinase gene *os-1*, the response regulator gene *rrg-1*, or the MAPKK gene *os-5*, (Figure S4). However, a circadian rhythm was observed in the histidine phosphotransferase *hpt-1* gene mRNA (p≤0.0001, period 23.3±0.8 h) and FLAG-tagged HPT-1 protein (HPT-1:FLAG) (p≤0.01, period 23.2±2.1 h) (Figure 5A & B). The FLAG tagged HPT-1 gene retained function, as a strain carrying the tag was viable (data not shown), unlike a strain carrying an *hpt-1* deletion [50].

**Figure 6:**
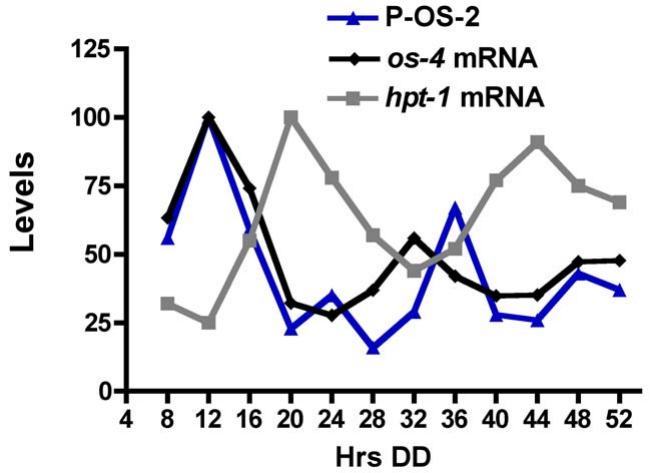
Comparison of the phases of OS-pathway component rhythms. Phospho-OS-2 protein (blue line and triangles; from Figure 4B with the peak set to 100), *hpt-1* mRNA (grey line and squares; from Figure 5A), and *os-4* mRNA (black line and diamonds; from Figure 2A with the peak set to 100) levels are plotted with the bars below the plot representing subjective day (white) and night (grey).

Interestingly, the peak time of accumulation for *hpt-1* mRNA is in the subjective early evening, antiphase to the peak in *os-4* mRNA and phospho-OS-2 levels (Figure 6 and, Vitalini et. al, 2007). As expected for a ccg, the rhythm in *hpt-1* mRNA accumulation was abolished in strains that lacked a functional FWO, with generally high levels of *hpt-1* mRNA accumulation at all times of day in the ΔFRQ strain, and low levels in the ΔWC-1 strain (Figure 5A). Similar to *os-4*, these data are consistent with positive regulation of *hpt-1* expression by the WCC; however, this regulation is likely not direct as the *hpt-1* gene was not found in ChIP-seq to be a direct target of the WCC [44]; no consensus WCC binding site is present in the *hpt-1* gene promoter, and the peak phase of *hpt-1* expression occurs when the WCC is inactive in cells grown in the dark [48].

### Physiological relevance of clock control of the OS pathway: kinetics of response

**Figure 7:**
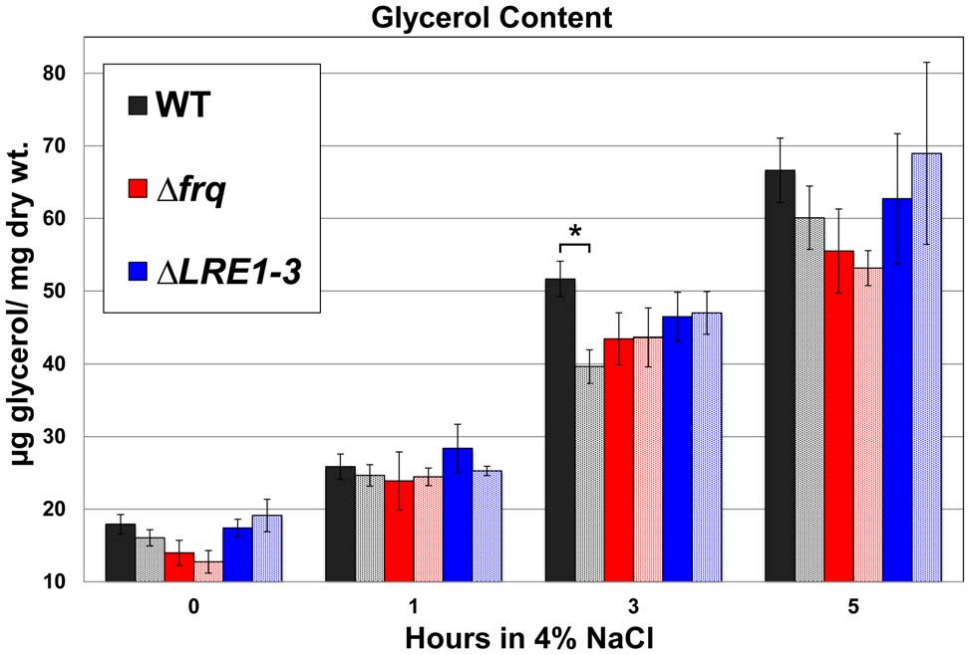
Clock-dependent kinetic advantage in glycerol accumulation following salt stress. The average concentration of glycerol (µg/mg tissue) is plotted from cultures grown in constant darkness (DD) and treated with 4% NaCl from 0 to 5 hours at two different times of day (DD12, solid bars, versus DD 24, stippled bars) in the indicated strains (± SEM; n = 4 to 8). The asterisk represents a statistically significant difference in the glycerol levels was observed in WT strains treated at DD12 and DD24 (p<0.001; n = 8 student T-test).

Activation of the OS pathway by a salt shock results in an increase in intracellular glycerol as an osmo-protectant [15], [16], [50]. Daily rhythms in phospho-OS-2 might be predicted to cause rhythms in glycerol levels in the absence of a stress. However, we observed no clear rhythms in glycerol levels over the course of the day. Alternatively, the levels of phospho-OS-2 observed at the circadian peak in the early morning may be lower or qualitatively different than the activation achieved by a salt shock. If this is true, we predicted that there would be a circadian difference in glycerol accumulation in response to osmotic stress. To test this possibility, WT Neurospora cells were treated at different circadian times with a salt shock, and glycerol production was monitored. As expected from our previous experiment, there was no difference in the glycerol content in subjective morning (DD12) or subjective evening (DD24) tissue at time 0 in any of the strains examined (Figure 7). After 1 h in 4% NaCl, although the levels of glycerol have begun to increase, there was no time-of-treatment-dependent difference in the glycerol response for any strain. By 3 h in 4% NaCl, the WT tissue treated at DD12 showed a significant increase in glycerol content compared to the DD24 treatment. A functional clock is required for this time-of-treatment difference in glycerol, as ΔFRQ strains lack this different response. In addition, the time-of-day effect of salt treatment on glycerol levels is abolished in ΔLRE strains, suggesting that high amplitude rhythms in OS-2 phosphorylation are required for this effect. Additionally, the ΔLRE strain inappropriately over-accumulates glycerol when treated for 3 h in the subjective evening (DD24) compared to the corresponding WT treatment (p = 0.035 N = 5). The levels of glycerol after 3 hrs of salt treatment in the *ΔLRE1-3* strain and in the clock mutant strain lie within the range observed in the WT strain; however unlike in the WT strain, they do not vary according to circadian time of treatment. By 5 h in 4% NaCl, the acute shock had overridden any circadian control, leading to similar glycerol values when treated at DD12 or DD24.

## Discussion

In this study, we discovered biological regulation of a MAPK pathway by direct clock transcriptional control. The circadian clock controls expression of MAPK pathway components rather than affecting post-transcriptional signaling via receptors and phosphorylation cascades. These data suggest that circadian changes in the cellular environment, while not activating the pathway via receptor mediated stress sensation, can prime the basal activity of the MAPK pathway. As clocks and MAPK pathways are both evolutionarily conserved, and responsive to similar stressors, clock control of MAPK activation is expected to be widespread. Consistent with this idea, rhythms in the phosphorylated form of ERK, JNK, and p38 MAPKs have been observed in several higher eukaryotic model systems [25], [51], [52], [53], [54], [55], [56], [57], [58]. However, only a few examples of MAPK activity regulation via transcriptional control of the upstream MAPKKK or MAPKK are known in plants and mammals [59], [60], [61], and this form of regulation has not been observed in fungi. Future studies may reveal that transcriptional regulation of MAPK pathway components is more biologically relevant than thought previously.

### Direct transcriptional regulation by the clock controls MAPK phosphorylation

We have demonstrated that the light responsive WCC regulates *os-4* (MAPKKK) transcription by direct promoter binding (Figure 1B). Not only does the WCC bind and induce *os-4* transcript in response to a light pulse, but also in DD the WCC rhythmically binds to the *os-4* promoter to drive rhythmic transcription (Figures 2 and 3). Three prospective WCC binding sites in the *os-4* promoter were identified (Figure 1A) and deleted (*Δ*LRE1-3-*os-4*) to test their role in light responses and circadian rhythm generation. This deletion rendered *os-4* expression unresponsive to a light pulse (Figure 1C). Furthermore, consistent with the function of WCC as the positive circadian element, the triple binding site mutant also eliminated the circadian rhythms in *os-4* mRNA (Figure 4A). Expression of *os-4* was reduced, but not abolished, suggesting that other promoter elements and transcription factors are responsible for basal transcription. We do not know whether the three binding sites play differential roles in light or circadian regulation like the proximal and distal LREs in the *frq* promoter [46], [62], but future studies can address that question. Finally, and most significantly, the triple WCC binding site deletion of *os-4* disrupted the rhythm in phosphorylated OS-2 MAPK (Figure 4B) and circadian regulation of glycerol induction kinetics (Figure 7). These observations support our conclusion that transcriptional control of the MAPK pathway components leads to rhythms in MAPK pathway sensitivity.

### Elucidating the mechanism of day/night regulation of the OS-MAPK pathway

**Figure 8:**
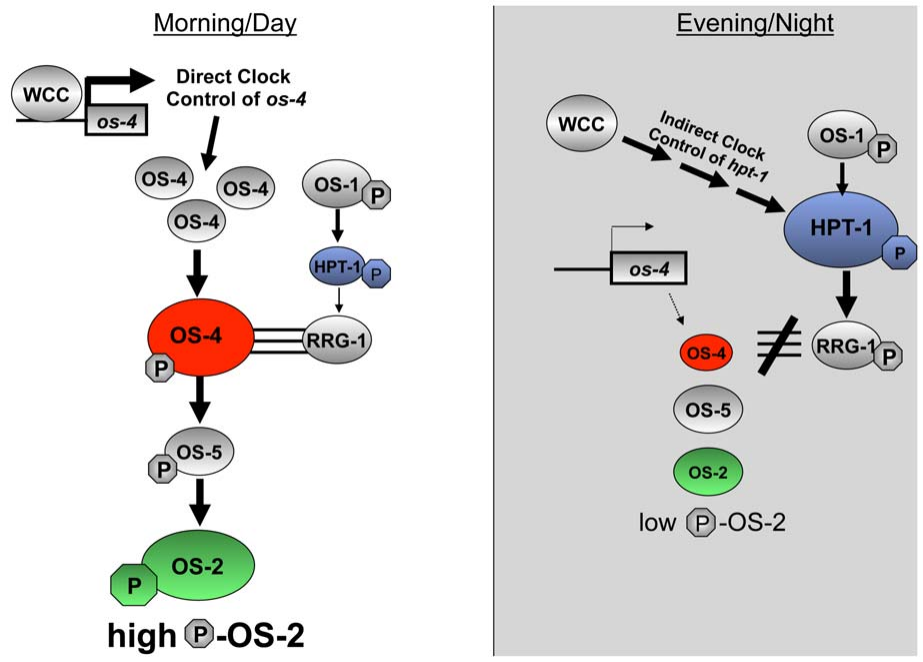
Model of circadian regulation of the OS MAPK pathway. See the text for details of the model. Single arrows refer to direct control and multiple arrows indicate indirect control. P = phosphorylation. Triple lines indicate physical interaction. The sizes of the circles for OS-4, HPT-1, and P-OS-2 reflect their relative levels during the day versus the night.

We have demonstrated that the FWO regulates transcript and protein levels of *os-4* and *hpt-1* (Figures 2 and 5). *os-4* is a direct clock target peaking in the subjective morning/day, and *hpt-1* is an indirect target peaking in the subjective evening/night. Like any biochemical pathway, the OS-pathway is governed by reaction rates and component equilibria. Circadian changes in the steady-state levels of the signaling components will shift the equilibria, and to lead to circadian changes in the pathway output. Our model (Figure 8) proposes that by controlling levels of OS-4 and HPT-1 proteins, the clock can tune the basal level of OS pathway activation in the absence of an osmotic shock. OS-4 is a positive regulator of phospho-OS-2, while HPT-1 is a predicted negative regulator of phospho-OS-2 through phosphorylation of RRG-1 [10], [19]. Because these two genes are expected to have opposite effects on the regulation of OS-2 activity, their anti-phase expression rhythms should synergize to activate OS-2 in the morning/day, and inactivate OS-2 in the evening/night (Figure 8). For instance, in the subjective morning, at DD12, OS-4 protein is at a peak, and low HPT-1 levels reduce the levels of RRG-1 phosphorylation, which is thought to increase RRG-1 interaction with OS-4 and increase MAPK activation. Both the decrease of HPT-1 and increase of OS-4 protein levels would shift the equilibrium of the unstressed pathway towards more basal signaling and would promote the phosphorylation of OS-2. Conversely, during the subjective night, at DD24, OS-4 is down-regulated while HPT-1 is up-regulated. An increase is HPT-1 would produce more phosphorylated RRG-1. Based on similarities to the yeast HOG pathway [13], phosphorylated RRG-1 is predicted to not physically interact with OS-4; thus, reducing MAPK pathway activation. At the same time, OS-4 protein is low. Therefore, in the night, the clock shifts the equilibrium of the pathway towards reduced basal signaling by increasing HPT-1 levels and decreasing OS-4 levels, and thus discourages phosphorylation of OS-2. Consequently, the dual clock inputs to the MAPK pathway work together to strengthen the day/night variation in basal MAPK activity.

Even though both inputs to the MAPK pathway are thought to contribute to the robustness of the rhythm in MAPK phosphorylation, disruption of just the direct input to *os-4* (ΔLRE1-3-*os-4* mutant) is sufficient to abolish the high amplitude phosphorylation rhythm in OS-2 (Figure 4) and the circadian regulation of glycerol induction kinetics in response to stress (Figure 7). This suggests a dominant role for rhythmic MAPKKK in generating rhythms in MAPK sensitivity. Evening-specific regulation of *hpt-1* could be achieved through indirect regulation from the WCC using a linear series of transcriptional activators (*i.e.* a morning-specific activator that is a direct target of the WCC activates another transcription factor that peaks later in the day, etc.), or through activation of a repressor by the WCC. However, our data support the first alternative; the levels of *hpt-*1 mRNA are low in the ΔWC-1 strain as compared to the WT strain, suggesting positive regulation of *hpt-1* by the clock. Experiments are currently underway to identify the transcription factor(s) that regulate *hpt-1* evening-specific transcription. Identification of this transcription factor(s) responsible for *hpt-1* mRNA rhythms will allow us to disrupt the binding site to determine if the rhythm in *os-4* transcription is sufficient for phospho-OS-2 rhythms, and if the rhythm in *hpt-1* contributes to the robustness of the phospho-OS-2 rhythm.

### Physiological consequences of the OS-2 phosphorylation rhythm

In response to stress dual threonine/tyrosine phosphorylation of a MAPK activates the kinase domain and brings about the full complement of downstream effects. We initially identified the OS-MAPK pathway as controlling the circadian expression of *ccg-1* [2], and one reasonable hypothesis would be that genes controlled by this pathway in response to osmotic stress might also be circadianly regulated. While there are other stress induced and circadian outputs of this pathway (ex. *ccg-9*, trehalose synthase), not all OS-pathway induced genes show rhythmic expression (TML and DBP, unpublished data). Furthermore, glycerol levels were constant in tissue harvested over the course of the day, suggesting the possibility that the in the absence of stress, the organism is using rhythmic gene expression to prepare for stress, but not necessarily completing the full physiological response to stress. In other words, there may be qualitative or quantitative differences in the output of the MAPK protein resulting from how the pathway is activated. Such differences may arise from modified MAPK protein interactions, feedback mechanisms, or through other factors controlling the activity of the MAPK in clock-driven versus acute osmotic activation of the pathway. The fact that there is a different kinetic response in glycerol production at the subjective morning compared to subjective night when Neurospora is treated with an osmotic stress (Figure 7), suggests that the circadian effect is one of priming the pathway, not necessarily fully activating the pathway. The clock is thus tuning the responsiveness of this pathway to be more sensitive in the morning and less sensitive in the evening. A strain that does not show these differences is not as well attuned to its environment, and therefore lacks this circadian advantage. However, in nature, environmental and clock inputs converge on signal transduction pathways simultaneously. The integration of transcriptional control and acute activation of signal transduction pathways likely coordinate to maximally activate the OS-pathway in the morning/day when osmotic stress is most damaging.

While the mechanism of the circadian oscillator transcriptional negative feedback loop is highly conserved within the eukaryotes, the core oscillator components and/or their roles in the oscillator vary among phyla [63], [64]. In contrast, the MAPK pathway components [65], and clock-associated kinases and phosphatases [59], are highly conserved within eukaryotes. These observations suggest that circadian clocks evolved in the context of existing MAPK signaling pathway kinases, and co-opted these pathways to control rhythmicity in target genes of the pathways that are already geared to respond to environmental signals that recur with a 24 h periodicity. This makes sense, as it would provide a simple mechanism to coordinately control sets of genes that allow the organism to anticipate and respond to the daily occurrence of a particular event. Furthermore, in view of the connection between the circadian clock and MAPK pathways, it is probably not coincidental that defects in the clock and in MAPK signaling pathways share many commonalities in human disease, including immune system defects, cardiovascular disease, and cancer [6], [7], [8], [9], [66]. Thus a complete understanding of how the circadian clock and MAPK pathways are integrated in cells, including the role of transcriptional regulation of MAPK components by the clock, is essential to begin to solve these important issues in human health.

## Materials and Methods

### Strains and Growth Conditions

Media for vegetative growth conditions and crossing protocols are described [67]. All strains contained the *ras-1^bd^* mutation, which clarifies the developmental rhythm on long growth tubes. For simplicity, these are referred to as wild type (WT) with respect to the clock throughout. Strains containing the *hph* cassette were maintained on Vogels minimal media supplemented with 200 µg/mL of hygromycin B (Calbiochem, Darmstadt, Germany). Stains containing the *bar* cassette were maintained on Vogels minimal media lacking NH_4_NO_3_ and supplemented with 0.5% proline and 200 µg/mL BASTA (Bayer). Time course experiments were conducted as described [68] with the following modifications: Media (1× Vogels salts, 0.5% arginine, 2% glucose, pH 6.0), synchronization (30°C lights on to 25°C dark (DD)), and shifting scheme. Liquid shaking cultures of mycelia were grown in constant light (LL) for a minimum of 4 h and transferred to DD on day 1 [for collection at DD 36, 40, 44, 48, 52], day 2 [for collection at DD 12, 16, 20, 24, 28, 32], day 3 [for collection at DD8], and harvested either at 9:00 a.m. (DD 12, 16, 20, 36, 40, 44) or 5:00 p.m. (DD 8, 24, 28, 32, 48, 52) on day 3. Tissue for RNA, protein, or ChIP analysis was harvested by flash freezing in liquid N_2_ at the indicated times in DD for each experiment.

### Strain Construction

The ΔLRE1-3-*os-4* strain (DBP 1276; *mat a*, *ras-1^bd^*, *os-4* ^Δ*LRE1-3*^) was obtained as a homokaryon by crossing the heterokaryon (*mat a, os-4* ^Δ*LRE1-3*^, Δ*wc-1::bar* ^+^, Δ*mus52::hph*) with FGSC 2489 (74-OR23-IV, *mat*A) to generate DBP 1245 (*mat a, os-4* ^Δ*LRE1-3*^). DBP 1245 was then crossed to FGSC 1858 (*mat A, ras-1^bd^*) to incorporate *ras-1^bd^*. The hetokaryotic parent was obtained by co-transforming FGSC 9568 (*mat a,* Δ*mus52::hph*) with a Δ*wc-1::bar^+^* deletion construct and an unmarked ΔLRE1-3 deletion construct (consisting of the genomic region found in LGI supercontig 1 nt 4450161-4447943, with the three LREs between nt 4449236–4449133 deleted). Proper integration of the ΔLRE1-3-*os-4* construct at the native locus was confirmed by PCR and sequencing. A strain (DBP 1207) carrying an ectopic copy of a quinic acid responsive promoter driving *os-4* expression was also generated to test if *os-4* overexpression would constitutively over-activate OS-2 phosphorylation as predicted, but it failed to drive overexpression of *os-4*. The OS-4::MYC strain (DBP 1176; *mat A*, *ras-1^bd^*, *OS-4::7xMYC*, *his-3^+^::bar^+^*) was obtained as a homokaryon after crossing DBP 1074 (*mat A, ras-1^bd^, OS-4::7xMYC, his-3^+^::bar^+^*, Δ*mus52::hph*) with FGSC 1859 (*mat a, ras-1^bd^*). DBP 1074 was obtained by co-transforming DBP 636 (*mat A, ras-1^bd^, his-3,* Δ*mus52::hph*) with pDBP409 (C-terminal MYC-tagged OS-4 construct, see below) and pBM61-bar^+^. Proper integration of the MYC-tagged OS-4 construct at the native locus was confirmed by PCR and sequencing confirmed that no mutations were introduced. The HPT-1::FLAG strain DBP 1167 (*mat a, ras-1^bd^, HPT-1::3xFLAG::hph*) was obtained as a homokaryon after crossing the heterokaryon DBP1072 (*mat a, HPT-1::3xFLAG::hph,* Δ*mus52::bar^+^*) with FGSC 1858 (*mat A, ras-1^bd^*). DBP1072 was obtained by transforming FGSC9719 (*mat a,* Δ*mus52::bar^+^*) with pDBP396 (C-terminally FLAG-tagged HPT-1 construct, see below). Integration of the FLAG-tagged HPT-1 construct was confirmed by detection of a ∼20 kDa FLAG tagged protein (HPT-1::FLAG predicted size = 19.4 kDa) in the transformants by western blot.

### Plasmid Construction

Plasmid pDBP409 contains 2.4 kb of the C-terminal end of *os-4* (ending one codon before the stop codon) linked in frame to a 7xMYC tag obtained from pMF276 [69] followed by 2.0 kb of the 3′ UTR of *os-4*. Sequencing of the plasmid insert revealed that the hybrid PCR deleted some of the MYC tags (7× MYC instead of 13× MYC), but no other mutations were observed. Plasmid pDBP396 has a pRS426 (high copy yeast URA3 plasmid) backbone carrying the following insert; 658 bp of the C-terminal end of *hpt-1* (ending one codon before the stop codon), an in frame 17× glycine linker, an in frame 3xFLAG-tag, the hygromycin B resistance gene, *hph*, flanked by *LoxP* sites, followed by 796 bp of the 3′UTR of *hpt-1*. Sequencing of the plasmid insert determined that recombination in yeast was uneven yielding extra glycines (17× Gly instead of 10× Gly), however, no other mutations were found.

### Nucleic Acid Isolation, Hybridization, and Sequencing

RNA was prepared as described [70] and transcripts were detected in Northern blots using a [α-^32^P]-UTP labeled anti-sense RNA probe for *os-4*, and a [α-^32^P]-dCTP labeled DNA probe for *hpt-1* [68].

### Protein Isolation and Western Blotting

To assay levels of OS-2 phosphorylation, protein was extracted as described [10] with the following modification: The extraction buffer used was 100 mM Tris pH 7.0, 1% SDS, 10 mM NaF, 1 mM PMSF, 1 mM sodium ortho-Vanadate, 1X HALT Protease Inhibitor Cocktail (Thermo Scientific, Waltham MA). Protein concentration was determined using NanoDrop spectroscopy (A_280_ of 1 = 1 mg/ml protein), and 50 µg of protein were boiled for 5 minutes in 1× Laemmli sample buffer. Samples were run on 10% SDS/PAGE gels and blotted to an Immobilon-P nitrocellulose membrane (Millipore, Billerica MA) according to standard methods. Phospho-OS-2 was detected by western blot using Mouse anti-phospho p38 primary (#9216 Cell Signaling, Beverley MA), and anti-Mouse-HRP secondary (#170-6516 BioRad, Hercules, CA) antibodies. Total OS-2 protein was detected by western blot with Rabbit anti-Hog1 primary (sc-9079 Santa Cruz Biotech, Santa Cruz, CA), and anti-Rabbit HRP secondary (#170-6515 BioRad, Hercules CA) antibodies. Immuno-reactivity was visualized on X-ray film (Phenix, Candler, NC) with Super Signal West Pico chemi-luminescence Detection (Thermo Scientific, Waltham, MA).

For detection of OS-4::MYC and HPT-1::FLAG proteins, extracts were prepared in buffer (50 mM HEPES pH 7.4, 137 mM KCl, 10% glycerol, 5 mM EDTA) [71], supplemented with 1X HALT protease inhibitors (Thermo Scientific, Waltham, MA), and phosphatase inhibitors: 20 mM β-glycerophosphate, 5 mM Sodium Flouride,1 mM Sodium Vanadate. For OS-4::MYC, 50 µg of total proteins were run on a 6% SDS-polyacrylamide gel, and the tagged protein detected by western blot using 9E10 Mouse anti-c-MYC primary (SC-40, Santa Cruz Biotechnology, Santa Cruz, CA), and anti-Mouse-HRP secondary (#170-6516, BioRad, Hercules, CA) antibodies. For HPT-1::FLAG, 50 µg of total proteins were run on a 12% SDS-polyacrylamide gel, and the tagged protein detected by western blot using Rabbit anti-FLAG primary (#2368, Cell Signaling Technology, Beverley, MA), and anti-Rabbit-HRP secondary (#170-6515, BioRad, Hercules, CA) antibodies. Detection for both proteins was by chemi-luminescence using Super Signal Pico detection (Thermo Scientific, Waltham MA).

### WC-2 ChIP

Chromatin immuno-precipitation (ChIP) was performed as described in [43], [72] with the following modifications. Dark grown liquid shake cultures were cross-linked in 1% (v/v) formaldehyde (Sigma, St. Louis, MO) for 30 min, and then quenched with 125 mM Glycine for 5 min. Tissue was then rinsed for 5 min in 1X TBS, excess liquid removed on paper towels, and then frozen in liquid N_2_. Tissue was crushed with mortar and pestle under liquid N_2_ and approximately 0.5 ml ground tissue was suspended in 1 ml lysis buffer (50 mM HEPES pH 7.5; 137 mM NaCl; 1 mM EDTA; 1% Triton X-100; 0.1% Na deoxycholate; 0.1% SDS; 1X HALT protease inhibitor cocktail). DNA was fragmented to an average size of 500–1000 bp by probe sonication (Branson Sonifier, microtip probe). Extracts were clarified by 10 min centrifugation at 14 K rpm, and a Bradford assay was performed on the supernatant. The protein concentration of all extracts was normalized to 2 mg/mL with lysis buffer. Extract (1 ml) was incubated with 2 µl anti-WC-2 antisera (a generous gift from Y. Liu) with gentle shaking overnight at 4°C. Protein G/agarose beads (50 µl) (GE Healthcare, UK) blocked with salmon sperm DNA and BSA were added and incubated at 4°C for 6 h with end over end rotation. Beads were successively washed for 5 min with low salt IC buffer (0.1% SDS; 1% Triton X-100; 2 mM EDTA; 20 mM Tris pH 8; 150 mM NaCl), high salt IC buffer (0.1% SDS; 1% Triton X-100; 2 mM EDTA; 20 mM Tris pH 8; 500 mM NaCl), LNDET buffer (0.25 M LiCl; 1% NP-40; 1% Na deoxycholate; 1 mM EDTA; 10 mM Tris pH 8), and twice with 1X TE buffer. To elute bound protein complexes, beads were suspended in 250 µl elution buffer (0.1 M NaHCO3; 1% SDS) and heated to 65°C for 15 min with periodic mixing. The supernatants from two elutions were pooled (500 µl total) and cross-links were reversed by the addition of 20 µl 5 M NaCl and incubation at 65°C for ≥6 h. To degrade residual protein, samples were incubated at 50°C for 1 h with 40 µg/ml of proteinase K. DNA was isolated by phenol/chloroform/iso-amyl alcohol extraction followed by ethanol precipitation using 20 µg of glycogen as a carrier. DNA was resuspended in 50 µl 1X TE and subsequently analyzed by quantitative or semi-quantitative PCR.

### Analysis of ChIP

Semi-quantitative PCR was used to examine the light-induced binding of the WCC to the *os-4* promoter. Primers used to detect genomic regions present in the immuno-precipitated DNA were as follows: for *os-4* (os4F 5′-AACCTGGTCAGAACGCATCATA-3′ and os4R 5′- GCCGGAAATGAGATGACGAA-3′); for *hpt-1* (hpt1F 5′-CTGTGCGAGTTCCTCCATGCCG-3′ and hpt1R 5′- GATGAGGCAACACAGCCTTGACG-3′); for *cpc-1* (cpc1F 5′-AAAACCCAGACACGTGGTTCTC′3 and cpc1R 5′-GGAGGGCTGACAGACGACTTC-3′). Up to two primers sets were multiplexed in a single PCR reaction. PCR cycles were as follows: 95°C for 2 min; 95°C for 15 sec.; 60°C for 30 sec.; 72°C for 30 sec. (step 2–4 repeated 25×); 72°C for 7 min. PCR products were separated on 8% acrylamide gels and visualized by ethidium bromide fluorescence under UV light.

Immuno-precipitated DNA from a rhythmic time series was analyzed by absolute quantitative PCR using Fast SYBR Green Mastermix and Fast 7500 Real-Time PCR System (Applied Biosystems, Carlsbad, CA). For each of the primer sets listed above, a standard to be used for absolute quantification was generated by amplifying a PCR product from genomic DNA that includes the genomic region to be analyzed. Primers used to generate standards were as follows: *os-4* (os4conF 5′-GACAATACGTCCTCTGCAGGATGTG-3′ and os4conR 5′-CTGACCTGCAGCATCGTGAC-3′); *hpt-1* (hpt1conF 5′-CCAGTTGAGTCAGATCGTTCAGGTG-3′ and hpt1conR 5′-CGTGCGTCTGATTCGCAGAAC-3′); *cpc-1* (cpc1conF 5′-CTCTACAGAATAGCGCGCGCATC-3′ and cpc1conR 5′-CGTGCGTCTGATTCGCAGAAC-3′). The standard PCR product was purified using the PCR Clean-up Kit (Qiagen, Valencia, CA) and quantitated using gel electrophoresis and ethidium bromide staining compared to a DNA sample of known concentration. The concentration of the standard was determined by densitometric comparison between the two samples.

### Glycerol Assay

Neurospora tissue was ground under liquid N_2_ and assayed for glycerol content using the Free Glycerol Reagent (Sigma, F6428) and Glycerol Standard Solution (Sigma G7793) protocol supplied by Sigma.

### Statistical Analysis

Nonlinear regression to fit the rhythmic data to a sine wave (fitting period, phase, and amplitude) and a line (fitting slope and intercept), as well as Akaike's information criteria tests to compare the fit of each data set to the 2 equations, were carried out using the Prism software package (GraphPad Software, San Diego, CA). The p values reflect the probability that, for instance, the sine wave fits the data better than a straight line. The student T-test was used to determine significance in the difference in levels of glycerol from 5 independent experiments. The error bars in all graphs represent SEM from at least 6 independent biological replicates.

## Supporting Information

Figure S1 ***os-4*** **mRNA rhythms persist in** ***rrg-1*** **mutants.** Northern blot assay detecting *os-4* mRNA. Cells from mutant strains were harvested at the indicated times in the dark (DD). The RRG-1 deletion strain KB1052 [10], obtained from Dr. Katherine Borkovich, and the *rrg-1* null mutant strain (rrg-1COP1-4) [2] have been previously described.
(TIF)

Figure S2 **Phospho-OS-2 is induced by salt treatment in the ΔLRE1-3-** ***os4*** **mutant.** Liquid cultures of the indicated strains were grown in 1× Vogels salts, 0.5% arginine, 2% glucose, pH 6.0, in LL for 24 h at 30°C and then transferred to DD at 25°C. After 24 h of growth in DD, the cultures were treated with 4% NaCl for the indicated times and Western blots for phospho-OS-2 and total OS-2 were performed.
(TIF)

Figure S3 ***os-4*** **mRNA is induced by salt treatment in the ΔLRE1-3-** ***os4*** **mutant.** Cultures were grown as described in Supplemental Figure S2 and *os-4* mRNA detected by northern blot.
(TIF)

Figure S4 ***os-1, os-5, and rrg-1*** **mRNA do not accumulate with a circadian rhythm.** RNA was prepared from tissue grown in the dark (DD) for the indicated times, and transcripts were detected on northern blots using [α-32P]-UTP labeled anti-sense RNA probes for *os-1*, *os-5*, and *rrg-1*. Densitometirc analysis of the northern blots are plotted as the level of mRNA over rRNA (n = 3, ±SEM).
(TIF)

## References

[1] Waskiewicz AJ, Cooper JA (1995). Mitogen and stress response pathways: MAP kinase cascades and phosphatase regulation in mammals and yeast.. Curr Opin Cell Biol.

[2] Vitalini MW, de Paula RM, Goldsmith CS, Jones CA, Borkovich KA (2007). Circadian rhythmicity mediated by temporal regulation of the activity of p38 MAPK.. Proc Natl Acad Sci U S A.

[3] Woelfle MA, Ouyang Y, Phanvijhitsiri K, Johnson CH (2004). The adaptive value of circadian clocks: an experimental assessment in cyanobacteria.. Curr Biol.

[4] Dodd AN, Salathia N, Hall A, Kevei E, Toth R (2005). Plant circadian clocks increase photosynthesis, growth, survival, and competitive advantage.. Science.

[5] Watanabe S, Yamashita K, Ochiai N, Fukumori F, Ichiishi A (2007). OS-2 mitogen activated protein kinase regulates the clock-controlled gene *ccg-1* in *Neurospora crassa*.. Biosci Biotechnol Biochem.

[6] de Paula RM, Lamb TM, Bennett L, Bell-Pedersen D (2008). A connection between MAPK pathways and circadian clocks.. Cell Cycle.

[7] Fu L, Lee CC (2003). The circadian clock: pacemaker and tumour suppressor.. Nat Rev Cancer.

[8] Cuenda A, Rousseau S (2007). p38 MAP-kinases pathway regulation, function and role in human diseases.. Biochim Biophys Acta.

[9] Han J, Sun P (2007). The pathways to tumor suppression via route p38.. Trends Biochem Sci.

[10] Jones CA, Greer-Phillips SE, Borkovich KA (2007). The response regulator RRG-1 functions upstream of a mitogen-activated protein kinase pathway impacting asexual development, female fertility, osmotic stress, and fungicide resistance in Neurospora crassa.. Mol Biol Cell.

[11] Posas F, Chambers JR, Heyman JA, Hoeffler JP, de Nadal E (2000). The transcriptional response of yeast to saline stress.. J Biol Chem.

[12] Zhang Y, Lamm R, Pillonel C, Lam S, Xu JR (2002). Osmoregulation and fungicide resistance: the Neurospora crassa os-2 gene encodes a HOG1 mitogen-activated protein kinase homologue.. Appl Environ Microbiol.

[13] Hohmann S (2002). Osmotic stress signaling and osmoadaptation in yeasts.. Microbiol Mol Biol Rev.

[14] Krantz M, Becit E, Hohmann S (2006). Comparative genomics of the HOG-signalling system in fungi.. Curr Genet.

[15] Pillonel C, Meyer T (1997). Effect of phenylpyrroles on glycerol accumulation and protein kinase activity of *Neurospora crassa*.. Pestic Sci.

[16] Fujimura M, Ochiai N, Ichiishi A, Usami R, Horikoshi K (2000). Sensitivity to phenylpyrrole pesticides and abnormal glycerol accumulation in os and cut mutant strains of Neurospora crassa.. Pestic Sci.

[17] Albertyn J, Hohmann S, Thevelein JM, Prior BA (1994). GPD1, which encodes glycerol-3-phosphate dehydrogenase, is essential for growth under osmotic stress in Saccharomyces cerevisiae, and its expression is regulated by the high-osmolarity glycerol response pathway.. Mol Cell Biol.

[18] Akhtar N, Blomberg A, Adler L (1997). Osmoregulation and protein expression in a pbs2delta mutant of Saccharomyces cerevisiae during adaptation to hypersaline stress.. FEBS Lett.

[19] Fujimura M, Ochiai N, Oshima M, Motoyama T, Ichiishi A (2003). Putative homologs of SSK22 MAPKK kinase and PBS2 MAPK kinase of Saccharomyces cerevisiae encoded by os-4 and os-5 genes for osmotic sensitivity and fungicide resistance in Neurospora crassa.. Biosci Biotechnol Biochem.

[20] Bardwell L (2006). Mechanisms of MAPK signalling specificity.. Biochem Soc Trans.

[21] Chang L, Karin M (2001). Mammalian MAP kinase signalling cascades.. Nature.

[22] Chen RE, Thorner J (2007). Function and regulation in MAPK signaling pathways: lessons learned from the yeast Saccharomyces cerevisiae.. Biochim Biophys Acta.

[23] Irmler S, Rogniaux H, Hess D, Pillonel C (2006). Induction of OS-2 phosphorylation in *Neurospora crassa* by treatment with phenylpyrrole fungicides and osmotic stress.. Pesticide Biochem Physiol.

[24] Lara-Rojas F, Sanchez O, Kawasaki L, Aguirre J (2011). Aspergillus nidulans transcription factor AtfA interacts with the MAPK SakA to regulate general stress responses, development and spore functions.. Mol Microbiol.

[25] Pizzio GA, Hainich EC, Ferreyra GA, Coso OA, Golombek DA (2003). Circadian and photic regulation of ERK, JNK and p38 in the hamster SCN.. Neuroreport.

[26] Zarubin T, Han J (2005). Activation and signaling of the p38 MAP kinase pathway.. Cell Res.

[27] Ferreiro I, Joaquin M, Islam A, Gomez-Lopez G, Barragan M (2010). Whole genome analysis of p38 SAPK-mediated gene expression upon stress.. BMC Genomics.

[28] Dunlap J, Loros J, DeCoursey P (2004). Chronobiology.

[29] Young MW, Kay SA (2001). Time zones: a comparative genetics of circadian clocks.. Nat Rev Genet.

[30] Schibler U (2006). Circadian time keeping: the daily ups and downs of genes, cells, and organisms.. Prog Brain Res.

[31] Kozma-Bognar L, Kaldi K (2008). Synchronization of the fungal and the plant circadian clock by light.. Chembiochem.

[32] Panda S, Hogenesch JB, Kay SA (2003). Circadian light input in plants, flies and mammals.. Novartis Found Symp.

[33] Jin X, Shearman LP, Weaver DR, Zylka MJ, de Vries GJ (1999). A molecular mechanism regulating rhythmic output from the suprachiasmatic circadian clock.. Cell.

[34] Ripperger JA, Shearman LP, Reppert SM, Schibler U (2000). CLOCK, an essential pacemaker component, controls expression of the circadian transcription factor DBP.. Genes Dev.

[35] Yoo SH, Ko CH, Lowrey PL, Buhr ED, Song EJ (2005). A noncanonical E-box enhancer drives mouse Period2 circadian oscillations in vivo.. Proc Natl Acad Sci U S A.

[36] Gekakis N, Staknis D, Nguyen HB, Davis FC, Wilsbacher LD (1998). Role of the CLOCK protein in the mammalian circadian mechanism.. Science.

[37] Munoz E, Brewer M, Baler R (2002). Circadian Transcription. Thinking outside the E-Box.. J Biol Chem.

[38] Talora C, Franchi L, Linden H, Ballario P, Macino G (1999). Role of a white collar-1-white collar-2 complex in blue-light signal transduction.. Embo J.

[39] Dunlap JC, Loros JJ, Colot HV, Mehra A, Belden WJ (2007). A circadian clock in Neurospora: how genes and proteins cooperate to produce a sustained, entrainable, and compensated biological oscillator with a period of about a day.. Cold Spring Harb Symp Quant Biol.

[40] Loros JJ, Dunlap JC, Larrondo LF, Shi M, Belden WJ (2007). Circadian output, input, and intracellular oscillators: insights into the circadian systems of single cells.. Cold Spring Harb Symp Quant Biol.

[41] Cha J, Huang G, Guo J, Liu Y (2007). Posttranslational control of the Neurospora circadian clock.. Cold Spring Harb Symp Quant Biol.

[42] Brunner M, Kaldi K (2008). Interlocked feedback loops of the circadian clock of Neurospora crassa.. Mol Microbiol.

[43] He Q, Liu Y (2005). Molecular mechanism of light responses in Neurospora: from light-induced transcription to photoadaptation.. Genes Dev.

[44] Smith KM, Sancar G, Dekhang R, Sullivan CM, Li S (2010). Transcription factors in light and circadian clock signaling networks revealed by genomewide mapping of direct targets for neurospora white collar complex.. Eukaryot Cell.

[45] Cuadrado A, Nebreda AR (2010). Mechanisms and functions of p38 MAPK signalling.. Biochem J.

[46] Froehlich AC, Liu Y, Loros JJ, Dunlap JC (2002). White Collar-1, a circadian blue light photoreceptor, binding to the frequency promoter.. Science.

[47] He Q, Cheng P, Yang Y, Wang L, Gardner KH (2002). White collar-1, a DNA binding transcription factor and a light sensor.. Science.

[48] Schafmeier T, Haase A, Kaldi K, Scholz J, Fuchs M (2005). Transcriptional feedback of Neurospora circadian clock gene by phosphorylation-dependent inactivation of its transcription factor.. Cell.

[49] Noguchi R, Banno S, Ichikawa R, Fukumori F, Ichiishi A (2007). Identification of OS-2 MAP kinase-dependent genes induced in response to osmotic stress, antifungal agent fludioxonil, and heat shock in Neurospora crassa.. Fungal Genet Biol.

[50] Banno S, Noguchi R, Yamashita K, Fukumori F, Kimura M (2007). Roles of putative His-to-Asp signaling modules HPT-1 and RRG-2, on viability and sensitivity to osmotic and oxidative stresses in Neurospora crassa.. Curr Genet.

[51] Sanada K, Hayashi Y, Harada Y, Okano T, Fukada Y (2000). Role of circadian activation of mitogen-activated protein kinase in chick pineal clock oscillation.. J Neurosci.

[52] Coogan AN, Piggins HD (2003). Circadian and photic regulation of phosphorylation of ERK1/2 and Elk-1 in thesuprachiasmatic nuclei of the Syrian hamster.. J Neurosci.

[53] Obrietan K, Impey S, Storm DR (1998). Light and circadian rhythmicity regulate MAP kinase activation in the suprachiasmatic nuclei.. Nat Neurosci.

[54] Nakaya M, Sanada K, Fukada Y (2003). Spatial and temporal regulation of mitogen-activated protein kinase phosphorylation in the mouse suprachiasmatic nucleus.. Biochem Biophys Res Commun.

[55] Hasegawa M, Cahill GM (2004). Regulation of the circadian oscillator in Xenopus retinal photoreceptors by protein kinases sensitive to the stress-activated protein kinase inhibitor, SB203580.. J Biol Chem.

[56] Hayashi Y, Sanada K, Fukada Y (2001). Circadian and photic regulation of MAP kinase by Ras- and protein phosphatase-dependent pathways in the chick pineal gland.. FEBS Lett.

[57] Hayashi Y, Sanada K, Hirota T, Shimizu F, Fukada Y (2003). p38 mitogen-activated protein kinase regulates oscillation of chick pineal circadian clock.. J Biol Chem.

[58] Williams JA, Su HS, Bernards A, Field J, Sehgal A (2001). A circadian output in Drosophila mediated by neurofibromatosis-1 and Ras/MAPK.. Science.

[59] Gallego M, Virshup DM (2007). Post-translational modifications regulate the ticking of the circadian clock.. Nat Rev Mol Cell Biol.

[60] Korotayev K, Chaussepied M, Ginsberg D (2008). ERK activation is regulated by E2F1 and is essential for E2F1-induced S phase entry.. Cell Signal.

[61] Doczi R, Brader G, Pettko-Szandtner A, Rajh I, Djamei A (2007). The Arabidopsis mitogen-activated protein kinase kinase MKK3 is upstream of group C mitogen-activated protein kinases and participates in pathogen signaling.. Plant Cell.

[62] Froehlich AC, Loros JJ, Dunlap JC (2003). Rhythmic binding of a WHITE COLLAR-containing complex to the frequency promoter is inhibited by FREQUENCY.. Proc Natl Acad Sci U S A.

[63] Loros JJ (1998). Time at the end of the millennium: the Neurospora clock.. Curr Opin Microbiol.

[64] Rosato E, Kyriacou CP (2001). Flies, clocks and evolution.. Philos Trans R Soc Lond B Biol Sci.

[65] Widmann C, Gibson S, Jarpe MB, Johnson GL (1999). Mitogen-activated protein kinase: conservation of a three-kinase module from yeast to human.. Physiol Rev.

[66] Gery S, Koeffler HP (2007). The role of circadian regulation in cancer.. Cold Spring Harb Symp Quant Biol.

[67] Davis RL, deSerres D (1970). Genetic and microbial research techniques for *Neurospora crassa*.. Methods Enzymol.

[68] Correa A, Lewis ZA, Greene AV, March IJ, Gomer RH (2003). Multiple oscillators regulate circadian gene expression in Neurospora.. Proc Natl Acad Sci U S A.

[69] Honda S, Selker EU (2009). Tools for fungal proteomics: multifunctional neurospora vectors for gene replacement, protein expression and protein purification.. Genetics.

[70] Bell-Pedersen D, Dunlap JC, Loros JJ (1996). Distinct cis-acting elements mediate clock, light, and developmental regulation of the Neurospora crassa eas (ccg-2) gene.. Mol Cell Biol.

[71] Garceau NY, Liu Y, Loros JJ, Dunlap JC (1997). Alternative initiation of translation and time-specific phosphorylation yield multiple forms of the essential clock protein FREQUENCY.. Cell.

[72] Johnson L, Cao X, Jacobsen S (2002). Interplay between two epigenetic marks. DNA methylation and histone H3 lysine 9 methylation.. Curr Biol.

