# Supplementary figures and images for: Direct Transcriptional Control of a p38 MAPK Pathway by the Circadian Clock in Neurospora crassa
Source: PLoS One. 2011 Nov 7;6(11):e27149. doi: 10.1371/journal.pone.0027149 (PMC3210137; doi:10.1371/journal.pone.0027149)

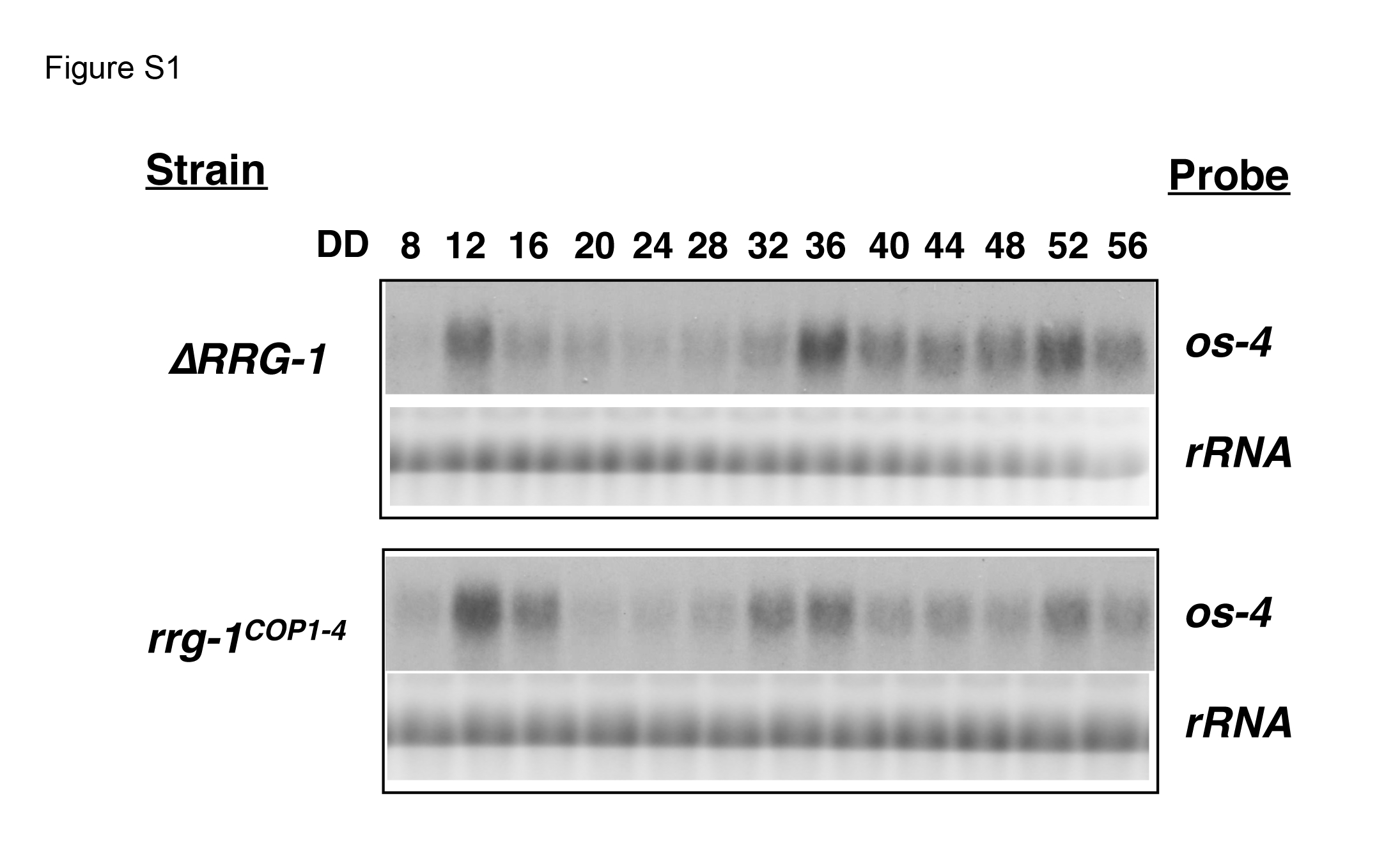

Supplement: Figure S1 — os-4 mRNA rhythms persist in rrg-1 mutants. Northern blot assay detecting os-4 mRNA. Cells from mutant strains were harvested at the indicated times in the dark (DD). The RRG-1 deletion strain KB1052 [10], obtained from Dr. Katherine Borkovich, and the rrg-1 null mutant strain (rrg-1COP1-4) [2] have been previously described. (TIF) [file pone.0027149.s001.tif]

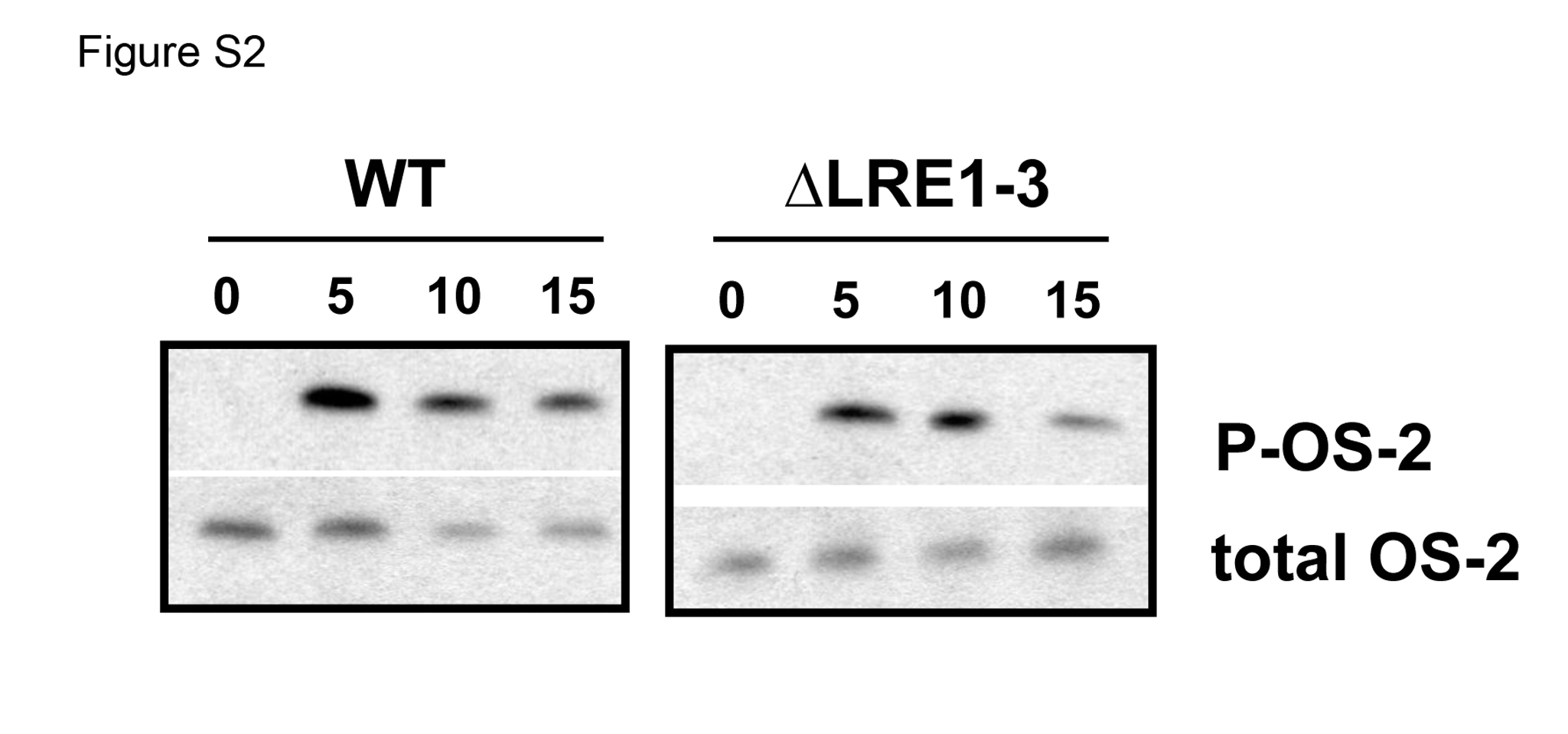

Supplement: Figure S2 — Phospho-OS-2 is induced by salt treatment in the ΔLRE1-3- os4 mutant. Liquid cultures of the indicated strains were grown in 1× Vogels salts, 0.5% arginine, 2% glucose, pH 6.0, in LL for 24 h at 30°C and then transferred to DD at 25°C. After 24 h of growth in DD, the cultures were treated with 4% NaCl for the indicated times and Western blots for phospho-OS-2 and total OS-2 were performed. (TIF) [file pone.0027149.s002.tif]

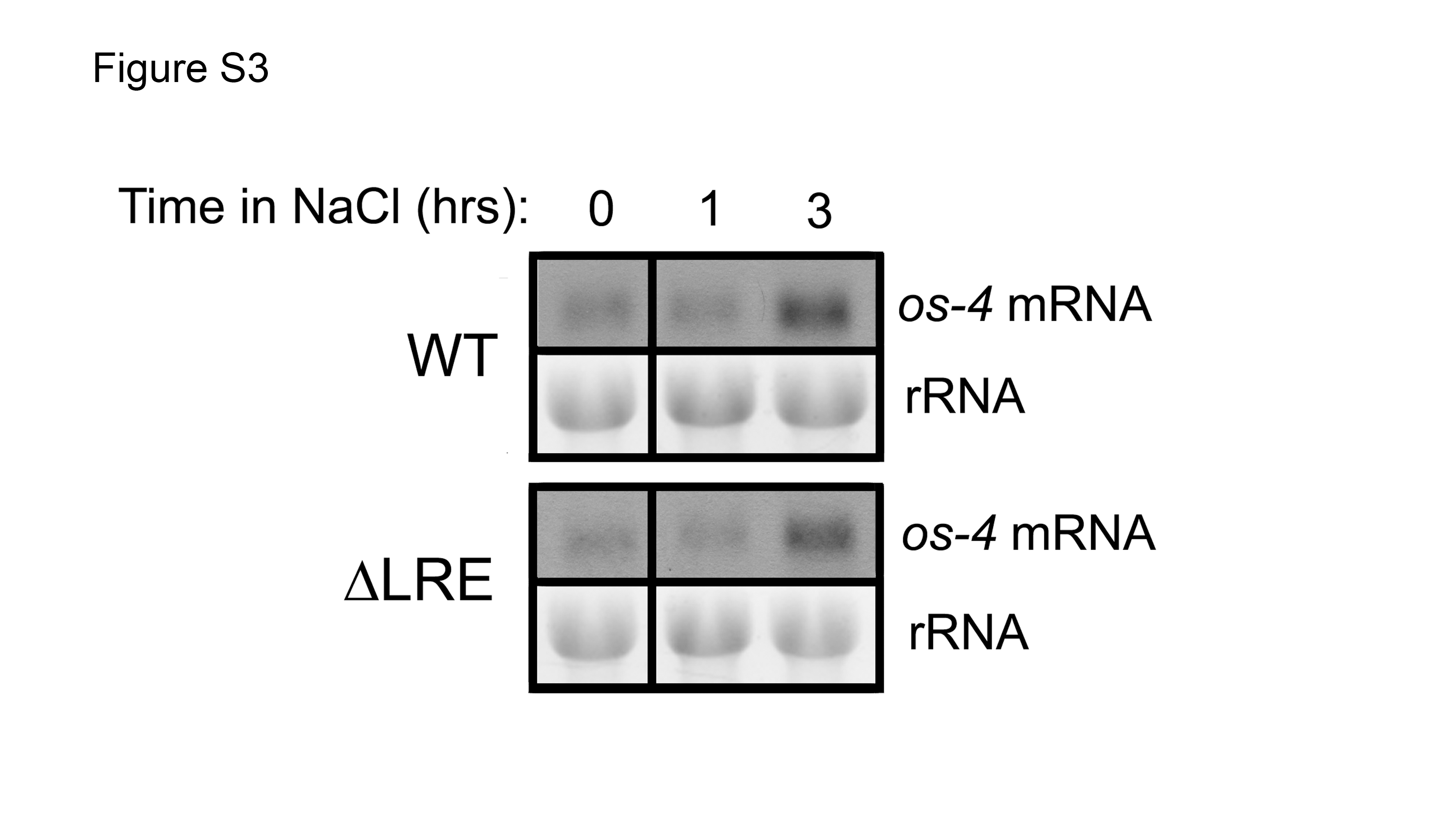

Supplement: Figure S3 — os-4 mRNA is induced by salt treatment in the ΔLRE1-3- os4 mutant. Cultures were grown as described in Supplemental Figure S2 and os-4 mRNA detected by northern blot. (TIF) [file pone.0027149.s003.tif]

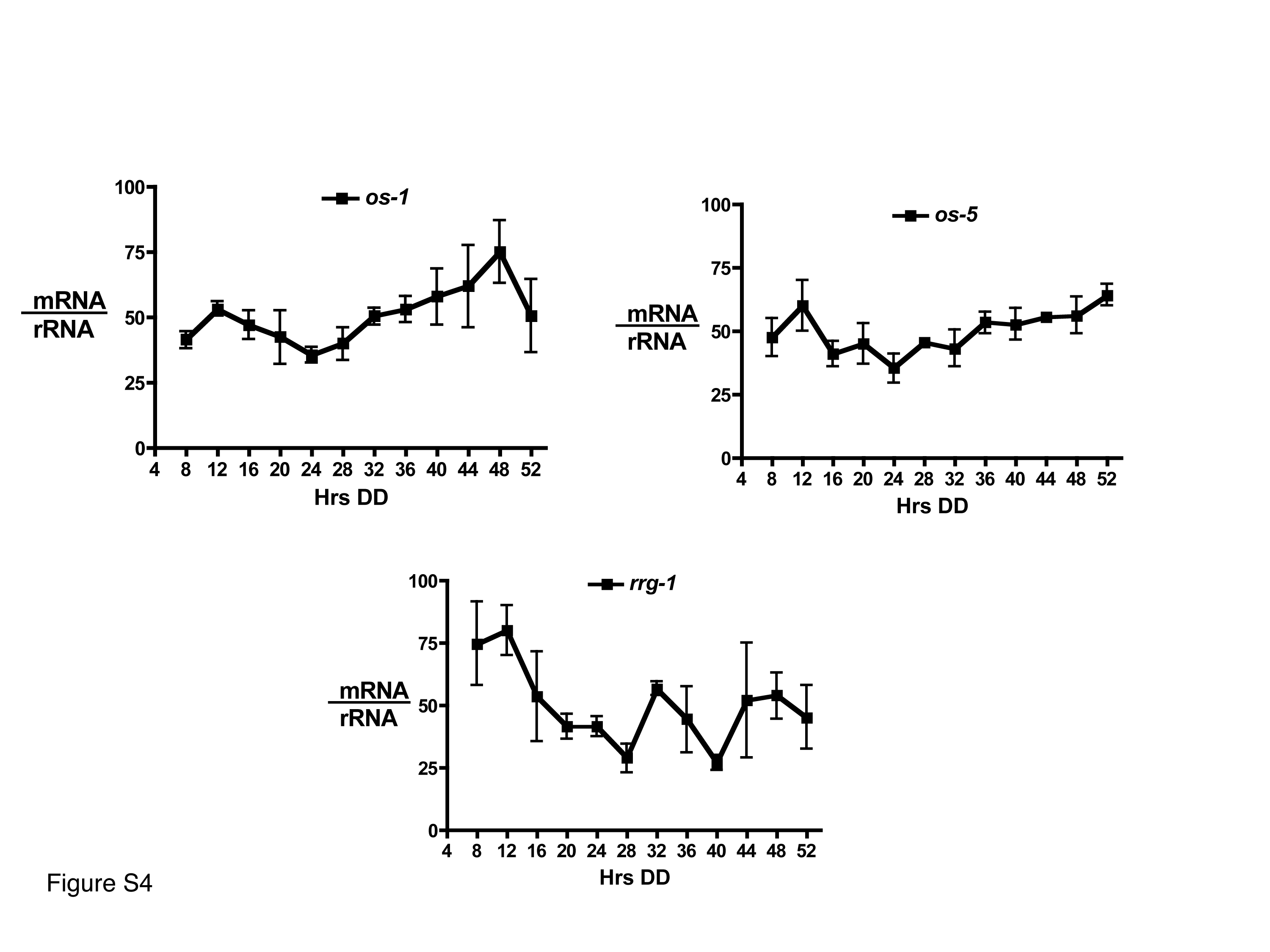

Supplement: Figure S4 — os-1, os-5, and rrg-1 mRNA do not accumulate with a circadian rhythm. RNA was prepared from tissue grown in the dark (DD) for the indicated times, and transcripts were detected on northern blots using [α-32P]-UTP labeled anti-sense RNA probes for os-1, os-5, and rrg-1. Densitometirc analysis of the northern blots are plotted as the level of mRNA over rRNA (n = 3, ±SEM). (TIF) [file pone.0027149.s004.tif]
